# Clindamycin use in head and neck surgery elevates the rate of infections in tracheostomies

**DOI:** 10.1007/s00405-022-07349-z

**Published:** 2022-03-25

**Authors:** Lukas S. Fiedler, Manuel Herbst, Hugo Pereira

**Affiliations:** 1 Department of Otorhinolaryngology and Head and Neck Surgery, University Medical Center Hamburg-Eppendorf, Hamburg, Germany; 2 Institute of Medical Biostatistics, Epidemiology and Informatics (IMBEI) Mainz, Department Biometry and Bioinformatics, Obere Zahlbacher Straße 69, 55131 Mainz, Germany; 3 Otorhinolaryngology and Head and Neck Surgery, Klinikum Mutterhaus der Borromäerinnen Mitte, Feldstraße 16, 54290 Trier, Germany

**Keywords:** Tracheostomy, SSI, Surgical wound infection, Antibiotic prophylaxis, Clindamycin

## Abstract

**Background:**

Surgical site infection (SSI) in open surgical tracheostomy (ST) occurs in up to 33% of the cases. SSI can be reduced by a postoperative antibiotic prophylaxis (POAP). The effect of Clindamycin on SSIs in head and neck surgery (HNS) is discussed controversially in the literature.

**Methods:**

An 8 year single-center retrospective comparative analysis of 441 STs (Visor-ST and Bjoerk-flap technique) performed within major HNS was evaluated due to the event of a SSI within 7 days and analyzed descriptively. Logistic regression model evaluated the impact of POAP with Clindamycin on SSIs.

**Results:**

The use of Clindamycin showed twice the rate of ST-SSI as all patients that did not receive Clindamycin, treated with other perioperative antibiotics. (Fisher’s *p* = 0.008) The logistic regression model could not prove a statistically significant impact. (OR = 2.91, *p* = 0.04).

**Conclusion:**

We recommend that Clindamycin should be reconsidered as a POAP regimen in ST. Further studies should evaluate alternatives for Penicillin-allergic patients.

**Level of evidence III:**

Comparative retrospective monocentric study.

## Introduction

The human and financial costs of treating surgical site infections (SSIs) are increasing [1]. A SSI is an infection originating in surgical wounds or the organs manipulated during an operative procedure, and is seen as the most common postoperative complication [2]. SSI occur within 30 days after the operation and consist of purulent drainage, organism growth from an aseptically won fluid or tissue, surgical wound exploration with no or a positive culture, or when a surgeon diagnoses a incisional wound infection [3].

The CDC classifies wounds according to the likelihood and degree of wound contamination during the operation. Therefore, wounds can be “clean”, where the respiratory, alimentary, genital, or uninfected urinary tract are not entered. “Clean-contaminated” wounds are operative wounds where the respiratory, alimentary, genital, or urinary tract is entered without unusual contamination. Most head and neck oncologic operations (HNOS) are counted to the group of “clean-contaminated” surgical wounds. Further, major breaks in sterile technique and incisions in which acute non-purulent inflammation is encountered, is classified as “contaminated wounds”, whereas “dirty or infected wounds” include necrotic wounds or perforated viscera. This last definition suggests that the organisms causing postoperative infection were present in the operative field before the operation [3].

The risk of establishing a SSI after head and neck surgery (HNS) ranges between a prevalence 0.37% [4], 20% [5] and 64% [6]. Lotfi et al. identified major risk factors for establishing a SSI undergoing HNOS as advanced stage cancer, smoking, comorbidities, the need of major reconstruction of the surgical wound, and the submission of an inadequate perioperative antibiotic prophylaxis (POAP) [7]. The use of POAP in clean-contaminated HNOS is seen as mandatory to reduce the risk of SSI. [6, 8–11] Successful POAP requires effect against gram-positive, gram-negative, and anaerobic organisms [12]. The highest prevention rate of HNS-SSI were found for Cefazolin, Amoxicillin-Clavulanate and Ampicillin-Sulbactam [6]. Further, Piperacillin-Tazobactam is a good choice of treatment as monotherapy for SSI after HNOS [13].

Within many HNOS, surgical tracheostomy (ST) remains the favorable technique to permanently or temporarily secure the airway [14]. Wound infection in open, elective ST is caused by the bacterial flora of the skin and occurs in up to 33% of the cases [15, 16]. This minor local infection may spread and can cause serious complications like tracheitis, mediastinitis, clavicular osteonecrosis and necrotizing fasciitis [17]. In ST and even in percutaneous dilatational tracheotomy, perioperative antimicrobial treatment showed a reduction of SSI [9, 18].

Studies identified Clindamycin use in postoperative clean-contaminated HNS as a risk factor for establishing a wound infection, whereas extended duration of antibiotics was found not to be associated with an increased risk of postoperative infection [6, 10, 12, 19]. Therefore, the choice of antibiotics seems to be more important than the duration of HNS POAP [20].

We concentrated on the evaluation of SSI after ST and tried to quantify the risk of wound infection in patients receiving Clindamycin compared with patients who received other antibiotics or none.

## Materials and methods

The work has been reported in line with the STROCSS criteria [21]. The trial has been registered under “Perioperative antimicrobial prophylaxis with Clindamycin elevates the rate of surgical site infections in tracheostomies? Findings in a retrospective comparative study in clean-contaminated head and neck surgery”, with researchregistry.com under “researchregistry7103”.

### Institutional review board review and data protection

The study is stated as exempt due to IRB approval and EU data protection regulations. Our retrospective chart review fits the exempt criteria. The research involves the collection of existing data, documents, records, pathological specimens or diagnostic specimens and the data is recorded in an anonymous manner such that subjects cannot be identified directly or through identifiers linked to the subject.

### Study population

The study population was derived from our electronic database of all consecutive patients who underwent ST (Visor-tracheostomy and Bjoerk-flap technique) in a primary ENT unit. Two groups of perioperative intravenous antimicrobial prophylaxis (Clindamycin 600 mg 3 × daily vs. other antibiotics) were compared due to occurred wound infections of the tracheostomy. Other antibiotics were Ceftriaxone 2 g (administered once/day), Piperazillin/Tazobactam 4/0,5 g (administered 3x/day), Cefuroxime 15 g (administered 3 × /day), Levofloxacin 500 mg (administered twice/day), Ampicillin/Sulbactam 3 g (administered 3 × /day), Meropenem 1,5 g (administered 3 × /day), Ciprofloxacin 400 mg (administered twice/day), and Vancomycin 1 g (administered twice/day) (see Table 1). The antimicrobial spectrum varies between those agents, therefore we summarized it for the most commonly used antibiotics in Table 2. The STs mostly conducted within HNOS between 2012 and 2020, were analysed. Experienced surgeons with training level “expert” (> 15years HNS) performed the STs within HNOS. Within 2012 and 2020 we analysed the quantity of ST-SSI in association with the POAP with Clindamycin. The ST-SSI were primarily diagnosed by ward doctors attended by consultants on their daily ward round. A ST-SSI was defined by peristomal redness and swelling with or without purulent secretion. Those criteria were applied to identify SSIs within our charts. Further, our “Tracheostomy care”-SOP (standard operating procedure) implicates the insertion of a PVC-based cuffed tube and dictates the first decannulation and tube change after 48–72 h after tracheostomy. This was constant over the observation period.

**Table 1:** Postoperative usage of antibiotics in ST

	*N* (%)
No antibiotic treatment	69 (15,65%)
Ceftriaxone	44 (9,98%)
Clindamycin	67 (15,19%)
Piperazillin/Tazobactam	226 (51,25%)
Cefuroxime	7 (1,59%)
Levofloxacin	1 (0,23%)
Ampicillin/sulbactam	17 (3,85%)
Meropenem	8 (1,81%)
Ciprofloxacin	1 (0,23%)
Vancomycin	1 (0,23%)
Total	**441** (**100%**)

**Table 2:** Antimicrobial coverage of most commonly used antibiotics [22, 23]

	Antimicrobial coverage
Ampicillin/sulbactam	Some Gram-positive (MSSA, Streptococcus), some Gram-negative, most Acinetobacter (sulbactam component has activity), excellent anaerobic activity
Clindamycin	Gram-positive, including ~ 50% of community-acquired MRSA, anaerobes but NOT Enterococci and NOT Gram-negative
Ceftriaxone	Gram-positive, enteric Gram-negative
Piperazillin/tazobactam	Gram positive, enteric Gram-negative, Pseudomonas, Anaerobes

### Data collection and statistical analysis

We retrospectively evaluated patient charts and operation protocols. Relevant data were collected: gender, age, surgical tracheostomy technique, postoperative antibiotic treatment and occurrence of tracheostomy site wound infections.

We counted and classified the postoperative wound infections within the first inpatient stay after the tracheostomy.

**Fig. 1:**
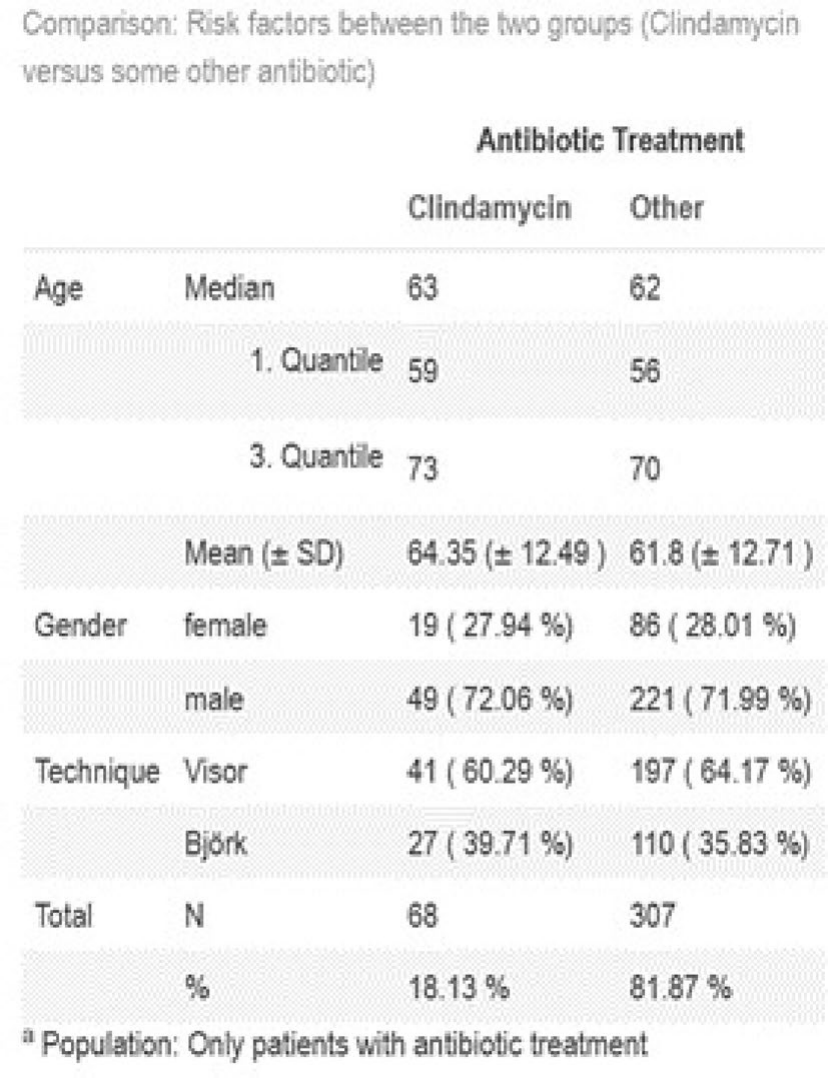
Risk factors establishing a SSI receiving antibiotics

In all patients where a wound infection occurred, antibiotics where used. We clustered the occurred wound infections in two groups Clindamycin use [yes; no] and other antibiotics used [yes; no] and evaluated the counts (see Fig. 1). The focus of our analysis lied on the question of whether Clindamycin or other antibiotics, did result in a higher quantity of ST-SSIs. We compared the risks of observing a SSI, first, without any adjustment for other potential risk factors, and in a second step, adjusting for age, gender, tracheostomy technique (Visor-tracheostomy vs. Bjoerk-flap technique) and Clindamycin use. In this second step, we chose a logistic regression model. In all steps, we estimated the Odds Ratio with the corresponding two sided 95%-confidence interval.

Reported *p* values were obtained by Fisher’s Exact Test for Count Data and, in case of logistic regression models, by the usual Wald-tests (*z* approximation). The level of significance is set to 5%.

We performed a purely descriptive data analysis using the software R version 4.1.1 (2021–01-30).

## Results

Our data set comprised a total of 453 tracheostomies, 161 were carried out using the Bjoerk-flap technique (BTS) and 292 were Visor-tracheostomies (VT). The majority (72.41%) of our patients was male. The mean age of the patients was 63 years (sd = 12.7 years). All our patients who established a ST-SSI received antibiotic treatment. The SSIs in ST occurred between the fourth on the eighth postoperative day. (MW_VT_ = 7 vs. MW_BTS_ = 6; 95% CI: [− 2, 3]).

### Risk of establishing a SSI

Due to a lack of recording we excluded 12 patients from our analysis. Of a total of 441 patients receiving a ST, wound infections occurred in 43% of all the cases, whereof a total of 14% of the patients received Clindamycin and 29% other antibiotics. (p = 0001) 156% overall patients did not receive POAP. After exclusion of the patients who did not receive POAP, 1813% overall received Clindamycin (see Fig. 1).

### Risk of establishing a SSI with clindamycin

**Fig. 2:**
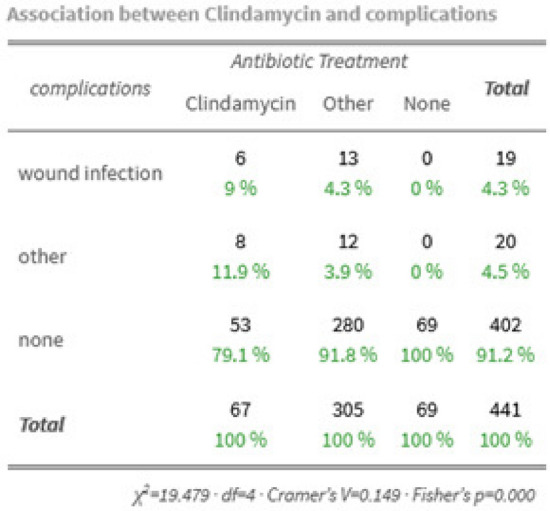
Does Clindamycin cause more wound infections in ST?

In question if Clindamycin cause more SSI -when looking at the ST collective receiving antibiotic treatment- we saw a statistically significant (Fisher’s *p* = 0008) two time-elevation (9% vs. 43%) of SSI in patients receiving Clindamycin compared with patients receiving other antibiotics (see Fig. 2). Our data analysis seems to support the hypothesis of Clindamycin being a risk factor for wound infection.

**Fig. 3:**
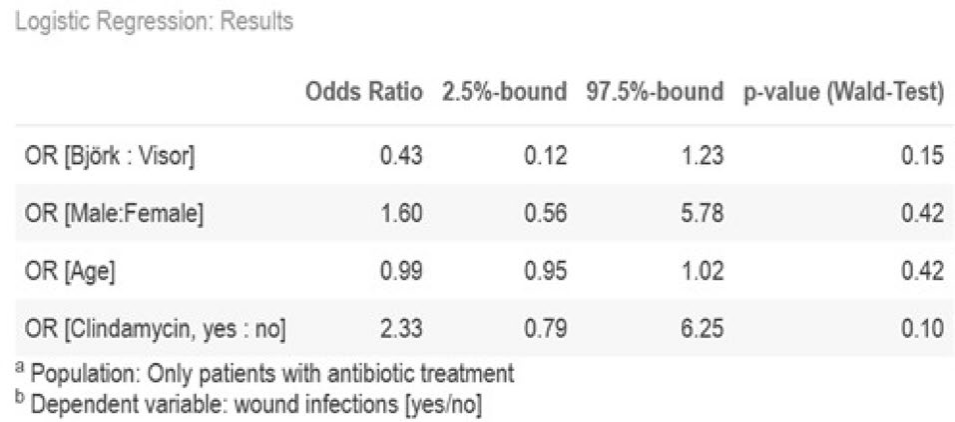
The influence of Clindamycin on wound infection in ST in patients receiving antibiotic treatment

Even though the effect is statistically significant, at this point we cannot conclude that Clindamycin is responsible for a higher SSI risk without considering other factors like gender, ST-technique and age. To this end we used a logistic regression model where the dependent variable wound infection is adjusted to gender, age and ST-technique. We found no statistically significant (Fisher’s p = 0,004) influence of Clindamycin on ST-SSI with an OR = 291 with two-sided 95% confidence interval [0.98; 7.79] (see Fig. 3).

Footnote: In the statistical analysis we could proof a *p* value of < 0.05, this would be statistically significant, but the 95% CI contains an OR = 1, hence the effect is not statistically significant. The reason is, that the p value und Confidence Intervals were computed using different methods. (95% CI Profile Likelihood Confidence Intervals; *p* value: Wald test *z* approximation).

To sum up, we did not find evidence against the hypothesis that Clindamycin POAP in major HNS or HNOS does not lead to a higher risk of SSI in ST.

## Discussion

Surgical tracheostomy (ST) within major head and neck oncologic operations (HNOS) remains the favorable technique to protect the airway and prevent from postoperative asphyxia [14]. As shown before, we used two techniques of ST, the Visor-tracheostomy (VT) and the Bjoerk-flap technique (BTS) (N_VT_ = 238 vs. N_BTS_ = 137). Surgical site infection (SSI) is seen as the most postoperative complication [2] and accounts for more than 20% of all healthcare-associated infections [4]. Further, the literature describes a SSI rate after clean-contaminated HNS ranging between 25 and 64% and of polymicrobial origin [6, 24]. In ST, this minor local infection may spread and can cause serious complications like tracheitis, mediastinitis, clavicular osteonecrosis and necrotizing fasciitis [17]. In our data, we showed a total of 43% SSI within our 441 ST patients. Hence this low rate of SSI in ST, POAP in general, seems to reduce the SSI-rate in ST performed within HNOS [9].

Given our data, SSI in ST mostly occurred within 7 days. As recommended by Bartella et al. the POAP for patients undergoing HNOS should be extended, because of a significantly decreased level of SSI in POAP until the fifth postoperative day [24]. All our patients received perioperative antibiotic treatment at least until the seventh postoperative day.

Given the antimicrobial spectre of gram-positive, gram-negative and anaerobic organisms [12], POAP in HNOS with a first generation Cephalosporine or Amoxicillin-Clavulanate and Ampicillin-Sulbactam are equally effective [11]. In our cases, we used Ceftriaxon (998%) as a third generation and Cefuroxime (158%) as a second generation Cephalosporine. Amoxicillin-Clavulanate was not administered within our study group. Ampicillin-Sulbactam (Unacid^©^) was used in 385% of the ST cases. Ampicillin-Sulbactam seems to be effective [11] to cover anaerobic microbes and further Piperacillin-Tazobactam, which we used in 5125% has been described with a positive effect on treating SSI in HNOS [13].

In our ST cases, a POAP with Clindamycin was administered in 1519%. Our data analysis supports the hypothesis of Clindamycin being a risk factor for SSI.

We could observe a statistically significant (Fisher’s *p* = 0,008) two time-elevated rate (9 vs. 43%) of SSI in patients receiving Clindamycin compared with patients receiving other antibiotics. However, we found no statistically significant influence of Clindamycin on ST-SSI. Even though we were not able to proof a statistically significant association, studies show that Clindamycin is less effective in avoiding SSI compared with Ampicillin-Sulbactam (Unacid^©^) in HNS [6].Some studies showed an even higher risk to establish SSI when Clindamycin is used as POAP in HNS [25, 26]. Even a self-reported β-lactam allergy is associated with an increased SSI risk mediated through receipt of alternate antibiotic prophylaxis [27]. For patients with a true penicillin allergy undergoing HNOS, studies recommend alternative antibiotics, such as cefuroxime, with a broad gram negative coverage [26]. We recommend to evaluate the SSI risk in ST patients receiving Clindamycin as POAP with a prospective study design to support our hypothesis. Further, a recommendation due to alternatives for Clindamycin in Penicillin-allergic patients should be given.

Our study had a few limitations. The retrospective study design -for one- underlays a prospective two-armed controlled clinical trial. Further, STs were performed by more than one surgeon (*N* = 6); therefore, minimal diverging techniques could have impacted the quantity of SSIs. In retrospective design there was no option to exactly identify the reason for Clindamycin use, for example an evident Penicillin-allergy. There could have been other reasons not to use other antibiotics, which could have impacted the statistical outcome. Due to design, we were not able to exclude previous radiation therapy, obese patients with short necks or patients with chronic uncontrolled diabetes. Hence, we cannot entirely rule out possible undetected sources of bias in our estimations and comparisons.

## References

[1] Berríos-Torres SI (2017). Centers for disease control and prevention guideline for the prevention of surgical site infection, 2017. JAMA Surg.

[2] Sumiyama Y, Arima Y (2002). Surgical site infection: SSI. Nihon Rinsho.

[3] Garner JS (1986). CDC guideline for prevention of surgical wound infections,1985. Supersedes guideline for prevention of surgical wound infections published in1982 (Originally published in November1985). Revis Infect Control.

[4] Al-Qurayshi Z (2019). Surgical site infection in head and neck surgery: a national perspective. Otolaryngol Head Neck Surg.

[5] Robbins KT (1990). Risk of wound infection in patients with head and neck cancer. Head Neck.

[6] Vander Poorten V (2020). Perioperative antibiotics in clean-contaminated head and neck surgery: a systematic review and meta-analysis. Adv Ther.

[7] Lotfi CJ (2008). Risk factors for surgical-site infections in head and neck cancer surgery. Otolaryngol Head Neck Surg.

[8] Simo R, French G (2006). The use of prophylactic antibiotics in head and neck oncological surgery. Curr Opin Otolaryngol Head Neck Surg.

[9] Sittitrai P, Siriwittayakorn C (2018). Perioperative antibiotic prophylaxis in open tracheostomy: a preliminary randomized controlled trial. Int J Surg.

[10] Vila PM, Zenga J, Jackson RS (2017). Antibiotic prophylaxis in clean-contaminated head and neck surgery: a systematic review and meta-analysis. Otolaryngol Head Neck Surg.

[11] Patel PN (2018). Evidence-based use of perioperative antibiotics in otolaryngology. Otolaryngol Head Neck Surg.

[12] Callender DL (1999). Antibiotic prophylaxis in head and neck oncologic surgery: the role of gram-negative coverage. Int J Antimicrob Agents.

[13] Rodrigo JP (2004). Efficacy of piperacillin-tazobactam in the treatment of surgical wound infection after clean-contaminated head and neck oncologic surgery. Head Neck.

[14] Fiedler LS (2021). A cartilage conserving concept of a surgical tracheostomy-introduction and analysis of safety and complications of the visor-tracheostomy-a retrospective monocentric comparative study over 8 years. Eur Arch Otorhinolaryngol.

[15] Cipriano A (2015). An overview of complications associated with open and percutaneous tracheostomy procedures. Int J Crit Illn Inj Sci.

[16] Goldenberg D (2000). Tracheotomy complications: a retrospective study of 1130 cases. Otolaryngol-Head and Neck Surg.

[17] Cheng E, Fee WE (2000). Dilatational versus standard tracheostomy: a meta-analysis. Ann Otol Rhinol Laryngol.

[18] Hagiya H (2014). Effects of antibiotics administration on the incidence of wound infection in percutaneous dilatational tracheostomy. Acta Med Okayama.

[19] Cannon RB (2017). Methods to reduce postoperative surgical site infections after head and neck oncology surgery. Lancet Oncol.

[20] Mitchell RM (2015). Antibiotic prophylaxis in patients undergoing head and neck free flap reconstruction. JAMA Otolaryngol Head Neck Surg.

[21] Agha R (2019). STROCSS 2019 guideline: strengthening the reporting of cohort studies in surgery. Int J Surg.

[22] Veve MP (2018). Multicenter assessment of antibiotic prophylaxis spectrum on surgical infections in head and neck cancer microvascular reconstruction. Otolaryngol Head Neck Surg.

[23] Medicine, S.S.o. (2011) Antibiotics Review https://errolozdalga.com/medicine/pages/OtherPages/AntibioticReview.ChanuRhee.html#top. Accessed 09 Feb 2022

[24] Bartella AK (2017). Prospective comparison of perioperative antibiotic management protocols in oncological head and neck surgery. J Craniomaxillofac Surg.

[25] Langerman A (2015). Laryngectomy complications are associated with perioperative antibiotic choice. Otolaryngol Head Neck Surg.

[26] Pool C (2016). Increased surgical site infection rates following clindamycin use in head and neck free tissue transfer. Otolaryngol Head Neck Surg.

[27] Lam PW (2020). Self-reported beta-lactam allergy and the risk of surgical site infection: a retrospective cohort study. Infect Control Hosp Epidemiol.

